# CXCL12 Induces Connective Tissue Growth Factor Expression in Human Lung Fibroblasts through the Rac1/ERK, JNK, and AP-1 Pathways

**DOI:** 10.1371/journal.pone.0104746

**Published:** 2014-08-14

**Authors:** Chien-Huang Lin, Chung-Huang Shih, Chih-Chieh Tseng, Chung-Chi Yu, Yuan-Jhih Tsai, Mauo-Ying Bien, Bing-Chang Chen

**Affiliations:** 1 Graduate Institute of Medical Sciences, College of Medicine, Taipei Medical University, Taipei, Taiwan; 2 School of Respiratory Therapy, College of Medicine, Taipei Medical University, Taipei, Taiwan; 3 Division of Pulmonary Medicine, Department of Internal Medicine, Taipei Medical University Hospital, Taipei, Taiwan; Casey Eye Institute, United States of America

## Abstract

CXCL12 (stromal cell-derived factor-1, SDF-1) is a potent chemokine for homing of CXCR4^+^ fibrocytes to injury sites of lung tissue, which contributes to pulmonary fibrosis. Overexpression of connective tissue growth factor (CTGF) plays a critical role in pulmonary fibrosis. In this study, we investigated the roles of Rac1, extracellular signal-regulated kinase (ERK), c-Jun N-terminal kinase (JNK), and activator protein-1 (AP-1) in CXCL12-induced CTGF expression in human lung fibroblasts. CXCL12 caused concentration- and time-dependent increases in CTGF expression and CTGF-luciferase activity. CXCL12-induced CTGF expression was inhibited by a CXCR4 antagonist (AMD3100), small interfering RNA of CXCR4 (CXCR4 siRNA), a dominant negative mutant of Rac1 (RacN17), a mitogen-activated protein kinase (MAPK) kinase (MEK) inhibitor (PD98059), a JNK inhibitor (SP600125), a p21-activated kinase inhibitor (PAK18), c-Jun siRNA, and an AP-1 inhibitor (curcumin). Treatment of cells with CXCL12 caused activations of Rac1, Rho, ERK, and c-Jun. The CXCL12-induced increase in ERK phosphorylation was inhibited by RacN17. Treatment of cells with PD98059 and SP600125 both inhibited CXCL12-induced c-Jun phosphorylation. CXCL12 caused the recruitment of c-Jun and c-Fos binding to the CTGF promoter. Furthermore, CXCL12 induced an increase in α-smooth muscle actin (α-SMA) expression, a myofibroblastic phenotype, and actin stress fiber formation. CXCL12-induced actin stress fiber formation and α-SMA expression were respectively inhibited by AMD3100 and CTGF siRNA. Taken together, our results suggest that CXCL12, acting through CXCR4, activates the Rac/ERK and JNK signaling pathways, which in turn initiates c-Jun phosphorylation, and recruits c-Jun and c-Fos to the CTGF promoter and ultimately induces CTGF expression in human lung fibroblasts. Moreover, overexpression of CTGF mediates CXCL12-induced α-SMA expression.

## Introduction

Idiopathic pulmonary fibrosis (IPF) is caused by chronic lung inflammation in response to unknown etiologic agents, leading to tissue destruction, fibroblast overgrowth, myofibroblast formation, and extracellular matrix (ECM) protein deposition, that result in severe respiratory insufficiency [1], [2]. The pathogenesis of IPF is poorly understood, and current therapies are ineffective [3]. Additionally, certain airway diseases, including chronic obstructive asthma, involve a significant degree of airway remodeling and pulmonary fibrosis [4], [5]. Resident fibroblasts are major regulator cells of ECM protein expression in connective tissues and are recruited to wound sites by the release of inflammatory mediators such as transforming growth factor-β (TGF-β), interleukin (IL)-8/CXCL8, and connective tissue growth factor (CTGF) [6]–[8]. Fibroblasts express no or only low levels of the CTGF, however, it is overexpressed during wound repair by fibrotic mediators such as TGF-β, thrombin, and endothelin-1 (ET-1) that contribute to the pulmonary fibrosis [5], [8], [9].

Chemokines are a group of small proteins (8∼14 kDa) involved in proinflammatory processes related to cell migration. Four subfamilies of chemokines are distinguished in terms of the position of their first two cysteine residues, CXC, CC, CX3C, and CXCL12/stromal cell-derived factor-1 (SDF-1), which are secreted by various cell types [10]. CXCL12 was first described as a factor produced by bone marrow stromal cells and is a potent chemoattractant for fibrocytes that contributes to pulmonary fibrosis [11], [12]. Moreover, CXCL12 has a pleiotropic function in developmental angiogenesis as well as hematopoietic myeloid and lymphoid cell homing and differentiation [13]–[16]. CXCL12 is a ligand of the chemokine receptor, CXCR4, and plays an important role in pulmonary fibrosis [17]. For example, a recent study indicated that bleomycin-induced pulmonary fibrosis in mice is blocked by the CXCR4 antagonist, AMD3100 [18]. A previous report demonstrated that CXCL12 activates CXCR4 to induce G protein-coupled signaling pathways, such as phosphoinositide 3-kinase (PI3K)/Akt, Rac1, Rho, mitogen-activated protein kinase (MAPK), and activator protein-1 (AP-1), which subsequently mediates cellular responses [19]–[22]. However, the roles of CXCL12 in regulating CTGF expression in lung fibroblasts and in fibroblast differentiation are currently unclear.

The CTGF belongs to the CCN family and is recognized as a key factor in pulmonary fibrosis [23]. The CTGF is not constitutively expressed in the resting stage of lung fibroblasts, but is overexpressed after stimulation by multiple profibrotic agents such as thrombin and TGF-β [8], [24]. Several studies demonstrated that elevated CTGF expression contributes to expressions of ECM proteins, cell migration, and the myofibroblastic phenotype in tissue repair [5], [24], [25]. Thus, CTGF overexpression plays a critical role in pulmonary fibrosis. The promoter region of the human *ctgf* gene contains many transcription factor binding sites including AP-1, signal transducer and activator of transcription (STAT), SMAD, basal control element-1 (BCE-1), nuclear factor-κB (NF-κB), specificity protein 1 (Sp1), and Ets-1 [26]–[28]. Our previous study indicated that activation of AP-1 contributes to thrombin-induced CTGF expression in human lung fibroblasts [8]. However, the role of AP-1 in regulating CTGF expression caused by CXCL12 in lung fibroblasts is still unknown.

Increasing lines of evidence have shown that Rac1 and extracellular signal-regulated kinase (ERK) mediate cell migration, chemotaxis, and expressions of inflammatory mediators such as intercellular adhesion molecule-1 (ICAM-1) in response to CXCL12 stimulation [29]–[31]. A previous study indicated that small G-binding proteins such as Rac1 induce ERK enzymatic activity [32]. Moreover, activation of ERK regulates transcription factor activity that ultimately controls expressions of profibrotic genes and contributes to pulmonary fibrosis [33]. For example, Rac1/ERK mediation of matrix metalloproteinase-9 (MMP-9) expression in alveolar macrophages is involved in pulmonary fibrosis [34]. CXCR4 is a G protein-coupled receptor that induces cell migration and extravasations in vivo through Rac activation in human HEP-G2 hepatoma cells [35]. CXCL12 induces Jurkat T cell migration through a CXCR4/Rac-dependent pathway [20]. Our previous study showed that thrombin-induced CTGF expression was dependent on c-Jun N-terminal kinase (JNK)/AP-1 activation [8]. Scupoli *et* *al.* (2008) reported that CXCL12 induced IL-8/CXCL8 production via a JNK/AP-1 pathway in human T-cell acute lymphoblastic leukemia [22]. Moreover, CXCL12-induced AP-1 activation and MMP-13 expression require ERK activation [21]. We previously showed that Rac1 and ERK are involved in thrombin-induced IL-8/CXCL8 expression in lung epithelial cells [36], [37]. However, the roles of Rac1, ERK, and JNK in regulating CXCL12-mediated AP-1 activation and CTGF expression in human lung fibroblasts are still unclear. In this study, we found that CXCL12, acting through CXCR4, activates the Rac1/ERK and JNK signaling pathways to induce AP-1 activation and CTGF expression in human lung fibroblasts. Moreover, CTGF mediates CXCL12-induced α-smooth muscle actin (α-SMA) expression and fibroblast differentiation.

## Materials and Methods

### Materials

CXCL12 (from *Escherichia coli*) was purchased from Peprotech (Rocky Hill, NJ, USA). AMD3100, SP600125, control small interfering RNA (con siRNA), and c-Jun siRNA (a mixture containing 2 specific c-Jun siRNAs) were purchased from Sigma (St. Louis, MO, USA). PD98059 and PAK18 were purchased from Calbiochem-Novabiochem (San Diego, CA, USA). Rac 1 expression construct sequences carrying the T17N (dominant negative, RacN17) mutation, Rac and Rho activity assay kits, and a chromatin immunoprecipitation (ChIP) assay kit were purchased from Upstate Biotech Millipore (Lake Placid, NY, USA). Minimum essential medium (MEM), fetal calf serum (FCS), penicillin/streptomycin, sodium pyruvate, L-glutamine, non-essential amino acids (NEAAs), Lipofectamine Plus reagent, and Lipofectamine 2000 reagent were purchased from Invitrogen Life Technologies (Carlsbad, CA, USA). CXCR4 siRNA (a mixture containing 4 specific CXCR4 siRNAs) was purchased from Qiagen (Valencia, CA, USA). CTGF siRNA (SMARTpool: a mixture containing 4 specific CTGF siRNAs) was purchased from GE Healthcare (Waukesha, WI, USA). An antibody specific for α-tubulin was purchased from Transduction Laboratories (Lexington, KY, USA). Antibodies specific for c-Jun phosphorylated at Ser63, c-Jun, ERK phosphorylated at Tyr204, ERK, CTGF, Rac1, c-Fos, and rabbit polyclonal IgG, and anti-mouse, anti-rabbit, and anti-goat IgG-conjugated horseradish peroxidase (HRP) were purchased from Santa Cruz Biotechnology (Santa Cruz, CA, USA). An antibody specific for α-SMA was purchased from Abcam (Cambridge, MA, USA). The human CTGF promoter (-747/+214) luciferase construct (pGL3-CCN2-Luc) was kindly provided by Dr. M.-L. Kuo (National Taiwan University, Taipei, Taiwan). The pcDNA was provided by Dr. M.-C. Chen (Taipei Medical University, Taipei, Taiwan). pBK-CMV-*Lac Z* (*LacZ*) was provided by Dr. W-W. Lin (National Taiwan University, Taipei Taiwan). All materials for sodium dodecylsulfate polyacrylamide gel electrophoresis (SDS-PAGE) were purchased from Bio-Rad (Hercules, CA, USA). All other chemicals were obtained from Sigma.

### Cell culture

A normal human embryonic lung fibroblast cell line (WI-38) was obtained from American Type Culture Collection (Manassas, VA, USA). Cells were grown in an MEM nutrient mixture, containing 10% FCS, 2 mM L-glutamine, 0.1 mM NEAAs, 1 mM sodium pyruvate, 50 U/ml penicillin G, and 100 µg/ml streptomycin, in a humidified 37°C incubator with 5% CO_2_. Cells were used between passages 18 and 28 for all experiments. After reaching confluence, cells were seeded onto 6-cm dishes for cell transfection, Rac and Rho activity assays, and immunoblotting, onto 10-cm dishes for the ChIP assay, and onto 12-well plates for the cell transfection and luciferase assays.

### Transfection and CTGF-luciferase assay

WI-38 cells (5×10^4^ cells/well) were seeded onto 12-well plates overnight. Cells were transfected the following day using Lipofectamine Plus with 0.5 µg of CTGF-Luc (−747/+214) and 0.5 µg of *Lac Z*. After 6 h, the medium was aspirated and replaced with basal medium devoid of FBS overnight, after which cells were stimulated with the CTGF (0∼30 ng/ml) for another 16 h before being harvested. Luciferase activity was determined with a luciferase assay system (Promega, Madison, WI, USA), and was normalized on the basis of *Lac Z* expression. The level of induction of luciferase activity was computed as the ratio of cells with and without stimulation.

### Western blot analysis

Western blot analyses were performed as described previously [38]. Briefly, WI-38 cells were either treated with the vehicle and CXCL12, pretreated with PD98059 for 30 min, or transfected with specific siRNAs as indicated using Lipofectamine 2000 for 24 h followed by CXCL12 treatment. Whole-cell lysates (30∼50 µg) were subjected to SDS-PAGE, and transferred onto polyvinylidene difluoride (PVDF) membranes which were then incubated in TBST buffer (150 mM NaCl, 20 mM Tris-HCl, and 0.02% Tween 20; pH 7.4) containing 5% non-fat milk. Proteins were visualized by specific primary antibodies and then incubated with HRP-conjugated secondary antibodies. Immunoreactivity was detected using enhanced chemiluminescence (ECL) following the manufacturer’s instructions. Quantitative data were obtained using a computing densitometer with a scientific imaging system (Eastman Kodak, Rochester, NY, USA).

### ChIP assay

ChIP assays were performed using a ChIP assay kit according to the manufacturer’s instructions. Briefly, WI-38 cells (4×10^7^ cells) were incubated with CXCL12 (10 ng/ml) for 20 min and then cross-linked with formaldehyde at 37°C for another 10 min. Cell lysates were sonicated and then centrifuged for 10 min at 15,000 ×*g* at 4°C to spin down the cell debris. Soluble cross-linked chromatins were immunoprecipitated with anti-c-Jun, anti-c-Fos, and anti-rabbit IgG antibodies. DNA was purified and eluted with 50 µl of elution buffer using a spinning filter. Polymerase chain reaction (PCR) amplifications of AP-1 response elements on the CTGF promoter region were performed using the following primers: AP-1, 5′-GGA TGT ATG TCA GTG GAC AGA-3′ (sense) and 5′-AAG CGC AGT ATT TCC AGC ACC-3′ (antisense). Extracted DNA (2 µl) was used for 40 cycles of amplification in 50 µl of reaction mixture under the following conditions: 95°C for 30 s, 62°C for 60 s, and 72°C for 30 s. The PCR products were analyzed by 2% agarose gel electrophoresis.

### Rac and Rho activity assays

Rac activity was determined as described previously [35]. Briefly, cells were washed twice with ice-cold phosphate-buffered saline (PBS), lysed in 1 ml of magnesium lysis buffer (MLB) (25 mM HEPES (pH 7.5), 150 mM NaCl, 5% igepal CA-630, 10 mM MgCl_2_, 5 mM EDTA, 10% glycerol, 10 µg/ml aprotinin, and 10 µg/ml leupeptin), and centrifuged at 14,000 x*g* for 30 min. For the Rac or Rho activity assay, the lysate (0.8 ml) was incubated with 5 µg of PAK1 p21-binding domain (PBD)-agarose or 20 µg of Rhotekin RBD-agarose, respectively, at 4°C overnight. Beads were washed three times with MLB and centrifuged at 8,000 x*g* for 5 min. Bound Rac or Rho proteins were then solubilized in 20 µl of 2x Laemmli sample buffer and quantitatively detected by Western blotting (12% SDS-PAGE) using mouse monoclonal anti-Rac or anti-Rho antibody with the ECL system.

### Immunofluorescence staining

WI-38 cells were plated onto 24-mm-diameter round coverslips in 6-well plates. After stimulation with CXCL12 (10 ng/ml) for 48 h, cells were fixed in 4% paraformaldehyde at 37°C for another 15 min. Slides were blocked with 5% normal calf serum and incubated with an anti-α-SMA antibody at 37°C for 18 h. Slides were washed in PBS and the immunoreactivity was visualized by incubating the slides with a secondary antibody conjugated with FITC (Santa Cruz Biotechnology) at 37°C for 1 h. Slides were counterstained with DAPI to visualize nuclei. Slides were then examined under a fluorescence microscope (BX50 Olympus, Tokyo, Japan).

### Actin stress fiber formation assay

WI-38 cells (5×10^3^ cells/well) were seeded onto 4 chamber culture slides in an MEM nutrient mixture containing 10% FCS. The next day, the medium was aspirated and replaced with fresh MEM devoid of FCS overnight. Cells were pretreated with AMD3100 (10 µM) for 20 min, and stimulated with CXCL12 (10 ng/ml) for another 24 h; cells were fixed in 4% paraformaldehyde at 37°C for another 10 min. Slides were blocked with 5% normal calf serum and incubated with rhodamine-phallodin (Cytoskeketon, Denver, Co, USA) for 1 h. Slides were counterstained with DAPI to visualize nuclei. Slides were then examined under a confocal fluorescence microscope (Leica TCS SP5, Wetzlar, Germany).

### Statistical analysis

Results are presented as the mean ± S.E. from at least three independent experiments. A one-way analysis of variance (ANOVA) followed by Dunnett’s test was used when appropriate to determine the statistical significance of the difference between means. Values of *p*<0.05 were considered statistically significant.

## Results

### CXCL12 induces CTGF expression

**Figure 1:**
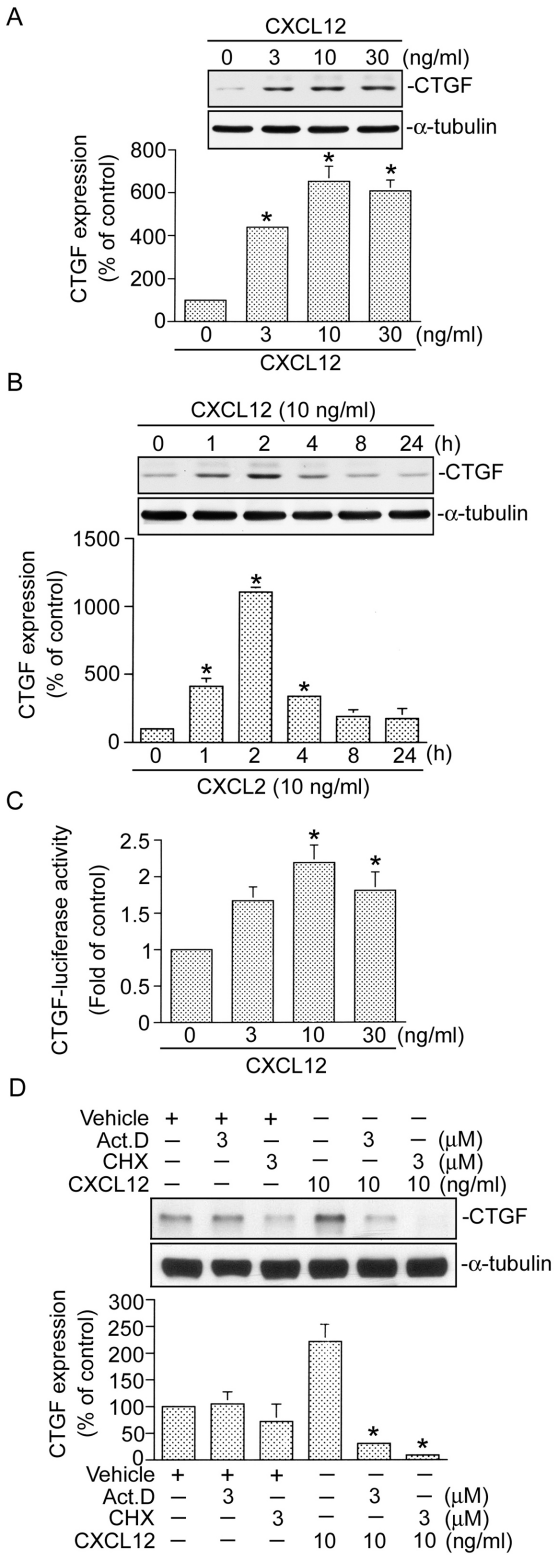
CXCL12 induces connective tissue growth factor (CTGF) expression in WI-38 cells. A, Cells were treated with various concentration of CXCL12 for 2 h. Whole-cell lysates were prepared and immunodetected with specific antibodies for the CTGF or α-tubulin. Data are presented as the mean ± S.E. of three experiments. *p<0.05, compared to the control group without CXCL12 treatment. B, Cells were treated with CXCL12 (10 ng/ml) for 0∼24 h, and then immunodetected with CTGF- or α-tubulin-specific antibodies. Data are presented as the mean ± S.E. of three experiments. *p<0.05, compared to the group without CXCL12 treatment. C, Cells were transfected with 0.5 µg of CTGF-Luc and 0.1 µg of pBK-CMV-Lac Z for 24 h and then stimulated with 3∼30 ng/ml of CXCL12 for another 16 h. Cells were harvested for a luciferase activity assay. Data are presented as the mean ± S.E. of four experiments. *p<0.05, compared to the group without CXCL12 treatment. D, Cells were pretreated with 3 µM actinomycin D (Act.D) and 3 µM cycloheximide (CHX) for 30 min and then stimulated with CXCL12 (10 ng/ml) for another 2 h. Whole-cell lysates were prepared and immunodetected with specific antibodies for the CTGF or α-tubulin. Data are presented as the mean ± S.E. of three experiments. *p<0.05, compared to CXCL12 treatment.

A previous study indicated that CXCL12 is a potent chemoattractant for fibrocytes that contributes to pulmonary fibrosis [11], [12]. The CTGF was recently identified as an agent in pulmonary fibrosis [5], [25], [26]. However, the connection between CXCL12 and the CTGF in human lung fibroblasts is still unclear. In this study, we investigated whether CXCL12 can induce CTGF expression in human lung fibroblasts (WI-38 cells). Incubation of WI-38 cells with CXCL12 (3∼30 ng/ml) for 2 h induced CTGF expression in a concentration-dependent manner, with a maximal effect at 10 ng/ml CXCL12 treatment (Fig. 1A). Moreover, this induction occurred in a time-dependent manner (Fig. 1B). After treatment, CTGF expression began to appear at 1 h, reached a maximum at 2 h, and then gradually decreased after 4 h (Fig. 1B). After 2 h of treatment with CXCL12 (10 ng/ml), CTGF expression had increased by 11.1±0.23-fold (Fig. 1B). In the following experiments, WI-38 cells were treated with 10 ng/ml of CXCL12 for 2 h. To further confirm whether CXCL12 can induce CTGF expression, WI-38 cells were transiently transfected with a human CTGF-Luc plasmid as an indicator of CTGF expression. As shown in Fig. 1C, treatment of WI-38 cells with CXCL12 (3∼30 ng/ml) induced an increase in CTGF-luciferase activity in a concentration-dependent manner. Treatment of cells with 10 ng/ml CXCL12 induced an increase in CTGF-luciferase activity by 2.2±0.24-fold (Fig. 1C). Next, to determine whether CXCL12-induced CTGF expression occurred through *de novo* synthesis, actinomycin D (a transcriptional inhibitor) and cyclohexamide (a translational inhibitor) were used. Treatment with 3 µM actinomycin D and 3 µM cyclohexamide both completely attenuated CXCL-12-induced CTGF protein expression (Fig. 1D). These results suggest that CXCL12-induced CTGF protein expression in WI-38 cells is dependent on *de novo* synthesis.

### CXCR4 mediates CXCL12-induced CTGF expression

**Figure 2:**
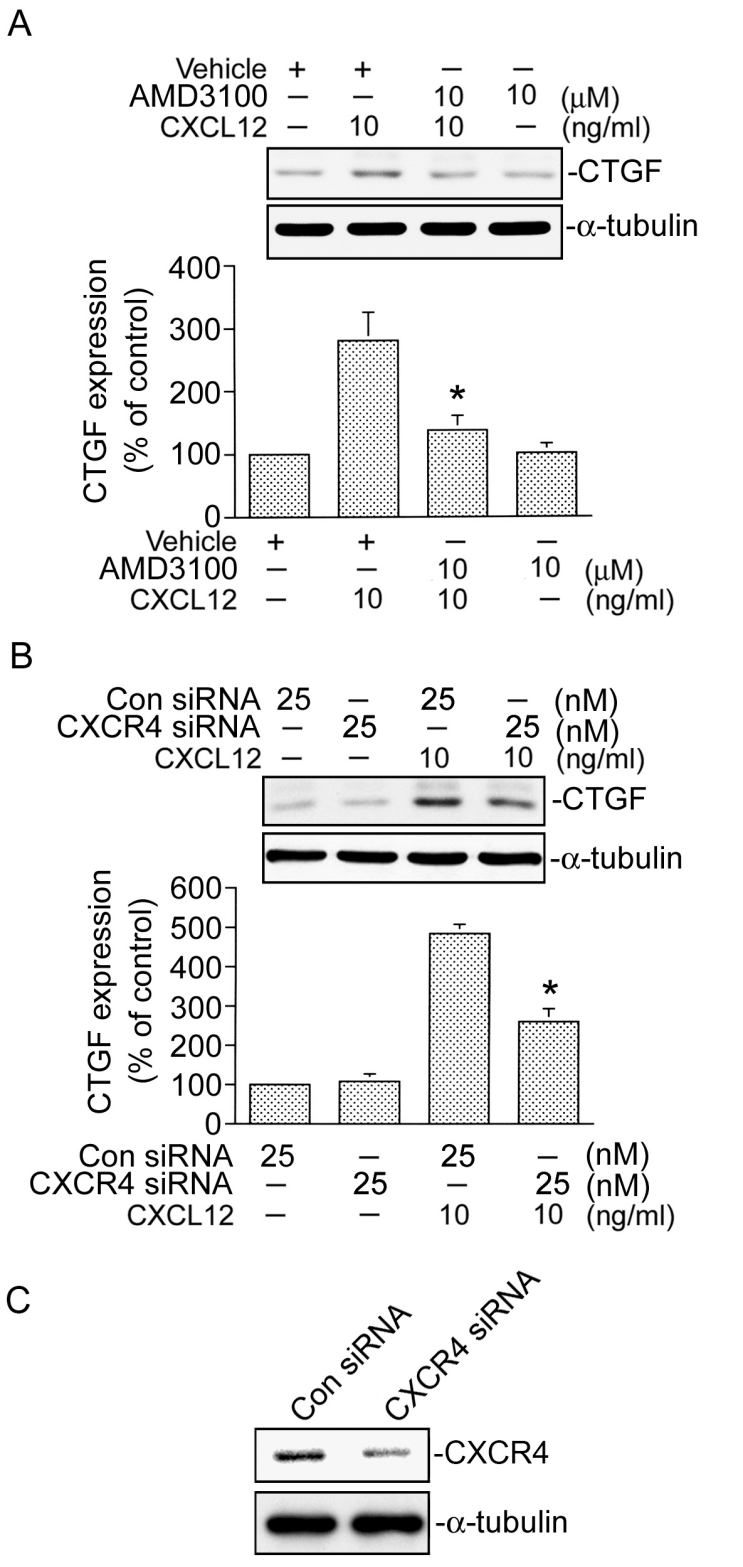
Involvement of CXCR4 in CXCL12 induces connective tissue growth factor (CTGF) expression in WI-38 cells. A, Cells were pretreated with 10 µM AMD3100 for 30 min and then stimulated with CXCL12 (10 ng/ml) for another 2 h. Whole-cell lysates were prepared and immunodetected with specific antibodies for the CTGF or α-tubulin. Data are presented as the mean ± S.E. of three experiments. *p<0.05, compared to CXCL12 treatment. B, Cells were transfected with control siRNA (Con siRNA 25 nM) or CXCR4 siRNA (25 nM) for 24 h and then stimulated with CXCL12 (10 ng/ml) for another 2 h. Whole-cell lysates were prepared and immunodetected with specific antibodies for the CTGF or α-tubulin. Data are presented as the mean ± S.E. of three experiments. *p<0.05, compared to CXCL12 stimulation. C, Cells were transfected with control siRNA (Con siRNA 25 nM) or CXCR4 siRNA (25 nM) for 24 h. Whole-cell lysates were prepared and immunodetected with specific antibodies for CXCR4 or α-tubulin. Traces represent results from three independent experiments.

A previous study demonstrated that CXCR4 is involved in CXCL12-induced cellular responses in various cells [20]. To identify whether CXCR4 is involved in CXCL12-induced CTGF expression in human lung fibroblasts, a CXCR4 antagonist (AMD3100) was used. Pretreatment of WI-38 cells with 10 µM AMD3100 markedly inhibited CXCL12-induced CTGF expression by 80±13% (*n* = 3) (Fig. 2A). Similarly, transfection of cells with CXCR4 siRNA (25 nM) suppressed CXCL12-induced CTGF expression by 60±8% (*n* = 3) (Fig. 2B). To further confirm result of the CXCR4 siRNA experiments, we also used CXCR4 siRNA to suppress CXCR4 protein expression. As shown in Fig. 2C, CXCR4 siRNA markedly inhibited CXCR4 expression in WI-38 cells (Fig. 2C). These results suggest that CXCR4 is involved in CXCL12-induced CTGF expression in WI-38 fibroblasts.

### Rac1 is involved in CXCL12-induced CTGF expression

**Figure 3:**
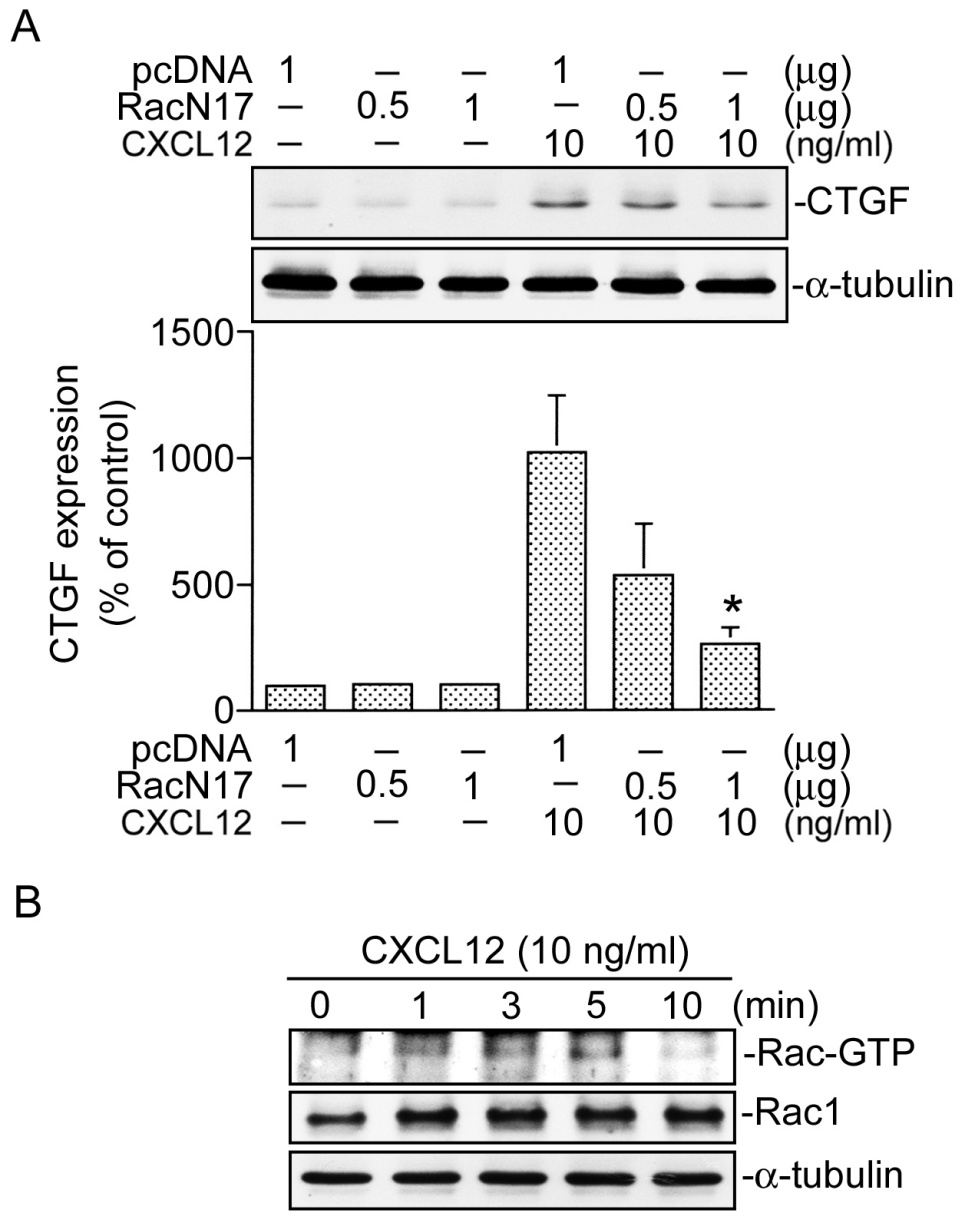
Involvement of Rac1 in CXCL12-induced connective tissue growth factor (CTGF) expression in WI-38 cells. A, Cells were transfected with pcDNA (1 µg) or RacN17 (0.5 and 1 µg) for 24 h and then stimulated with CXCL12 (10 ng/ml) for another 2 h. Whole-cell lysates were prepared and immunodetected with specific antibodies for the CTGF or α-tubulin. Data are presented as the mean ± S.E. of three experiments. *p<0.05, compared to CXCL12 stimulation. B, Cells were treated with CXCL12 (10 ng/ml) for 0∼10 min. Whole-cell lysates were then immunoprecipitated with PAK-1 PBD-agarose. The Rac activity assay is described in “Materials and Methods”. Rac1 and α-tubulin protein levels were determined in total lysates by a Western blot analysis as the loading control. Immunoblots are representative of three experiments with similar results.

A previous report demonstrated that small G proteins, including Rac1, are involved in the signaling pathway leading to induction of protein expression [36]. To investigate the role of Rac1 in mediating CXCL12-induced CTGF expression in WI-38 cells, RacN17 was used. We found that transfection of cells with RacN17 (0.5 and 1 µg) inhibited CXCL12-induced CTGF expression in a concentration-dependent manner, while 1 µg of RacN17 reduced CXCL12-induced CTGF expression by 83±7% (*n* = 3) (Fig. 3A). To further reveal whether Rac1 activation is involved in the signaling pathway of CXCL12-induced CTGF expression, Rac activity was measured after CXCL12 stimulation. Treatment of cells with CXCL12 (10 ng/ml) induced an increase in Rac activity in a time-dependent manner, as assessed by immunoblot samples for Rac immunoprecipitated from lysates using PAK1 PBD-agarose. The response had begun at 1 min and was sustained to 5 min after CXCL12 treatment (Fig. 3B). These results suggest that activation of Rac1 is involved in CXCL12-induced CTGF expression in human lung fibroblasts.

### ERK activation is involved in CXCL12-induced CTGF expression

**Figure 4:**
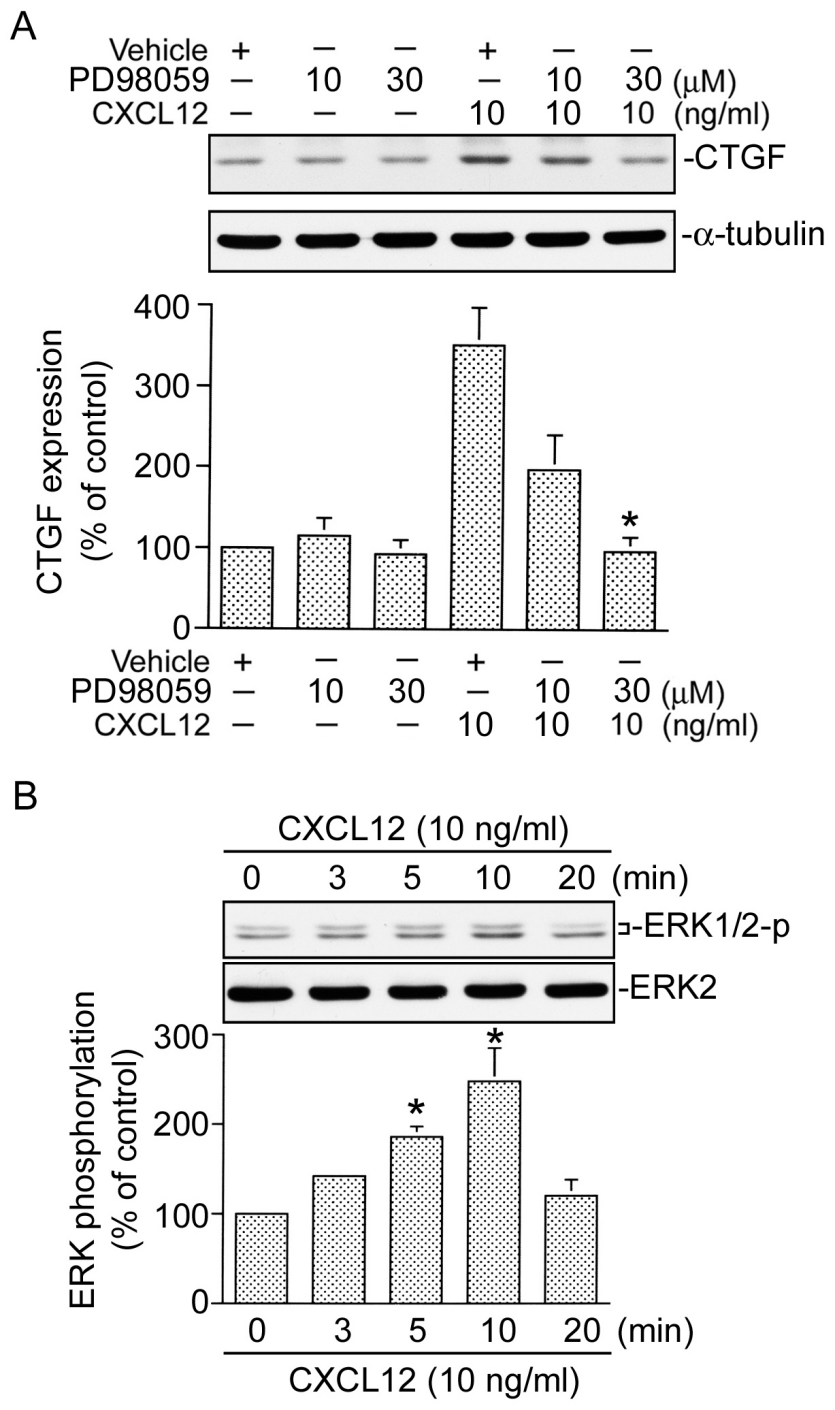
Involvement of extracellular signal-regulated kinase (ERK) in CXCL12-induced connective tissue growth factor (CTGF) expression in WI-38 cells. A, Cells were pretreated with 10 and 30 µM PD98059 for 30 min and then stimulated with CXCL12 (10 ng/ml) for another 2 h. Whole-cell lysates were prepared and immunodetected with specific antibodies for the CTGF or α-tubulin. Data are presented as the mean ± S.E. of three experiments. *p<0.05, compared to CXCL12 treatment. B, Cells were treated with CXCL12 (10 ng/ml) for 0∼20 min. Whole-cell lysates were prepared and immunodetected with specific antibodies for phospho-ERK Tyr 204 or ERK2. Data are presented as the mean ± S.E. of three experiments. *p<0.05, compared to the control without CXCL12 stimulation.

**Figure 5:**
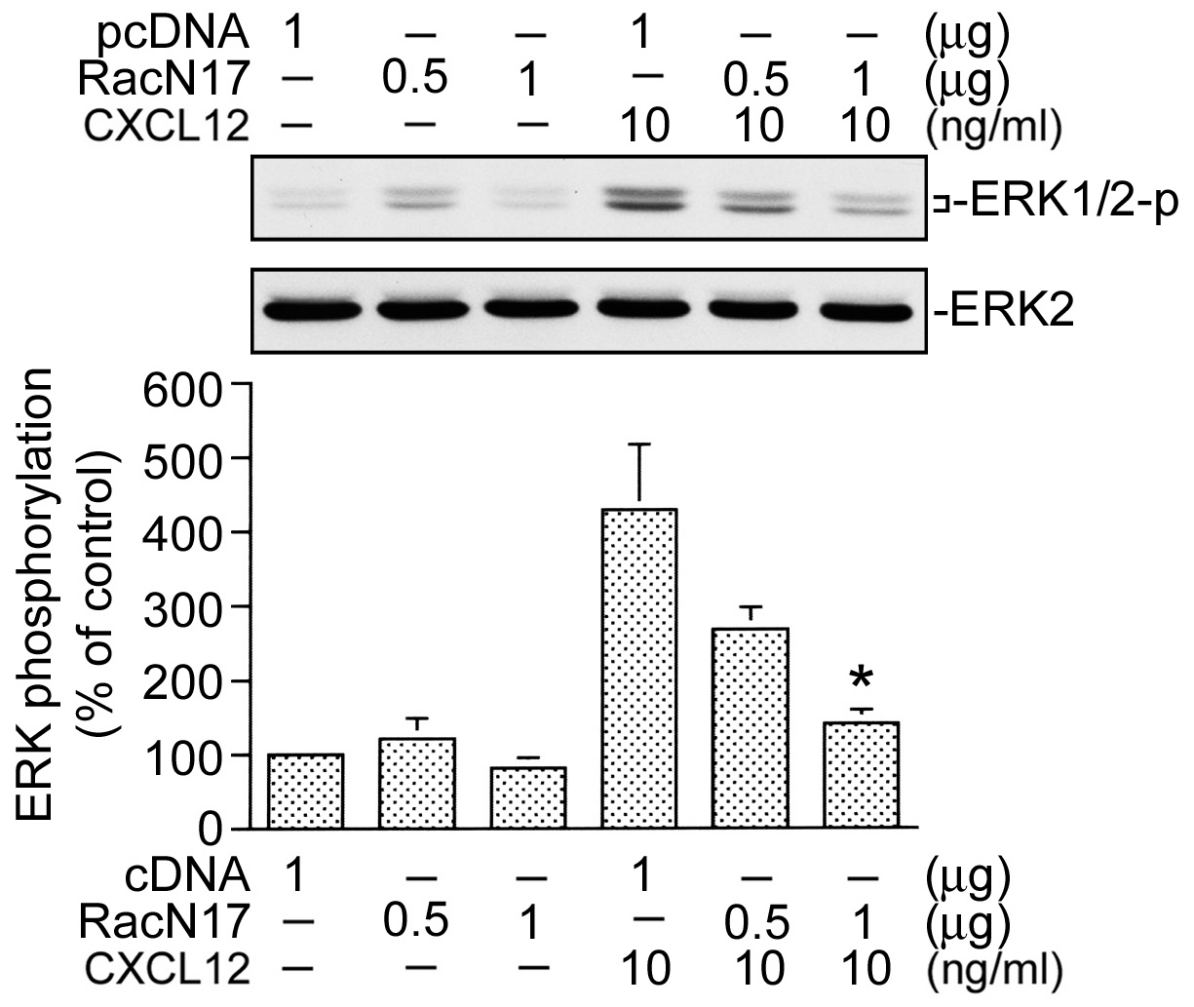
Involvement of Rac1 in CXCL12-induced extracellular signal-regulated kinase (ERK) phosphorylation in WI-38 cells. Cells were transfected with pcDNA (1 µg) or RacN17 (0.5 and 1 µg) for 24 h and then stimulated with CXCL12 (10 ng/ml) for another 10 min. Whole-cell lysates were prepared and immunodetected with specific antibodies for phospho-ERK Tyr 204 or ERK2. Data are presented as the mean ± S.E. of three experiments. *p<0.05, compared to CXCL12 stimulation.

Rac1 may activate a number of signal pathways including ERK [34]. To examine whether ERK activation is involved in the signal pathway leading to CTGF expression caused by CXCL12, PD98059 (an MEK inhibitor) was used. Pretreatment of cells with PD98059 (10 and 30 µM) inhibited CXCL12-induced CTGF expression. Treatment of cells with 30 µM PD98059 almost completely inhibited CXCL12-induced CTGF expression (Fig. 4A). We further examined whether CXCL12 is able to activate ERK. Because tyrosine phosphorylation of residue 204 in ERK causes enzymatic activation [21], an antibody specific against phosphorylated ERK Tyr204 was used. Treatment of WI-38 cells with 10 ng/ml CXCL12 resulted in an increase in ERK Tyr204 phosphorylation in a time-dependent manner. The response peaked at 10 min, and declined after 20 min of treatment (Fig. 4B, upper panel). The protein level of ERK was not affected by CXCL12 treatment (Fig. 4B, lower panel). These results suggest that ERK is involved in CXCL12-induced CTGF expression in WI-38 cells. Furthermore, transfection of cells with RacN17 (0.5 and 1 µg) inhibited CXCL-12-induced ERK phosphorylation in a concentration-dependent manner. When cells were treated with RacN17 (1 µg), CXCL12-induced ERK phosphorylation was inhibited by 82±6% (*n* = 3) (Fig. 5). Taken together, these results indicate that ERK is a downstream molecule of Rac1 in CXCL12-induced CTGF expression in WI-38 cells.

### p21-activated kinase (PAK) is involved in CXCL12-induced CTGF expression

**Figure 6:**
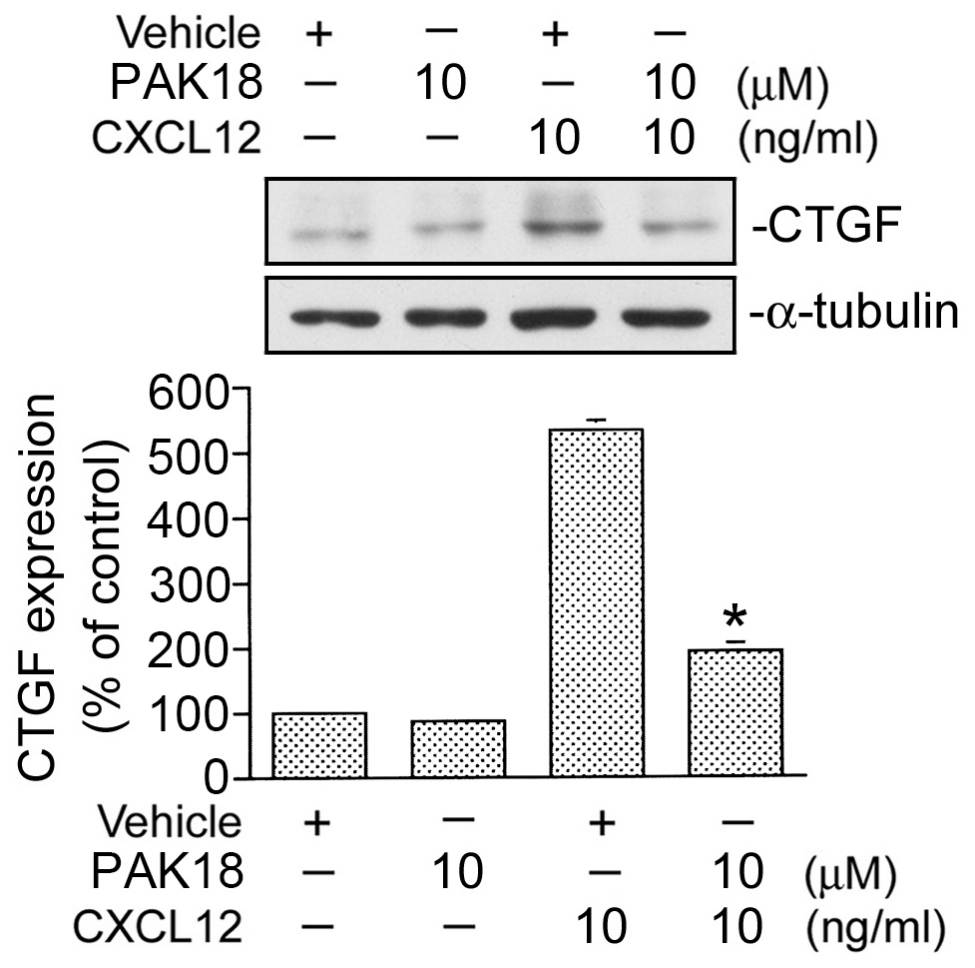
Involvement of p21-activated kinase (PAK) in CXCL12-induced connective tissue growth factor (CTGF) expression in WI-38 cells. Cells were pretreated with PAK18 (10 µM) for 30 min and then stimulated with CXCL12 (10 ng/ml) for another 2 h. Whole-cell lysates were prepared and immunodetected with specific antibodies for the CTGF or α-tubulin. Data are presented as the mean ± S.E. of three experiments. *p<0.05, compared to CXCL12 treatment.

A recent report demonstrated that PAK, a candidate for a downstream protein of Rac, plays a critical role in stretch-induced CTGF expression in glomerular mesangial cells [39]. To determine whether PAK is involved in CXCL12-induced CTGF expression, a PAK inhibitor (PAK18) was used. We found that PAK18 (10 µM) significantly inhibited CXCL12-induced CTGF expression by 75±2% (n = 3) (Fig. 6). This result suggests that PAK also participates in CXCL12-induced CTGF expression in WI-38 cells.

### AP-1 mediates CXCL12-induced CTGF expression

**Figure 7:**
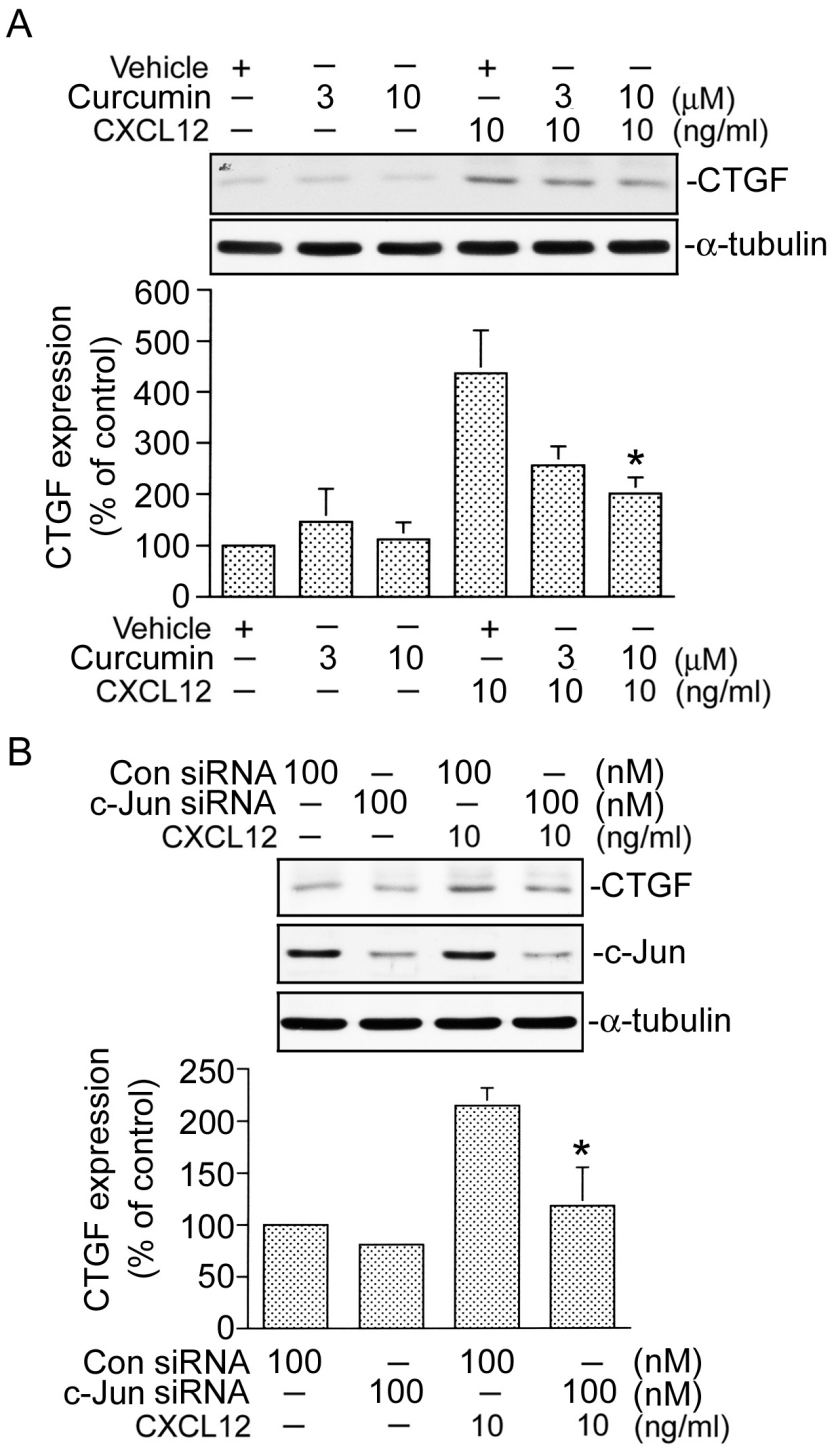
Involvement of activator protein-1 (AP-1) in CXCL12-induced connective tissue growth factor (CTGF) expression in WI-38 cells. A, Cells were pretreated with 3 and 10 µM curcumin for 30 min and then stimulated with CXCL12 (10 ng/ml) for another 2 h. Whole-cell lysates were prepared and immunodetected with specific antibodies for the CTGF or α-tubulin. Data are presented as the mean ± S.E. of four experiments. *p<0.05, compared to CXCL12 stimulation. B, Cells were transfected with control siRNA (Con siRNA 100 nM) or c-Jun siRNA (100 nM) for 24 h and then stimulated with CXCL12 (10 ng/ml) for another 2 h. Whole-cell lysates were prepared and immunodetected with specific antibodies for the CTGF, c-Jun, or α-tubulin. Data are presented as the mean ± S.E. of four experiments. *p<0.05, compared to CXCL12 stimulation.

**Figure 8:**
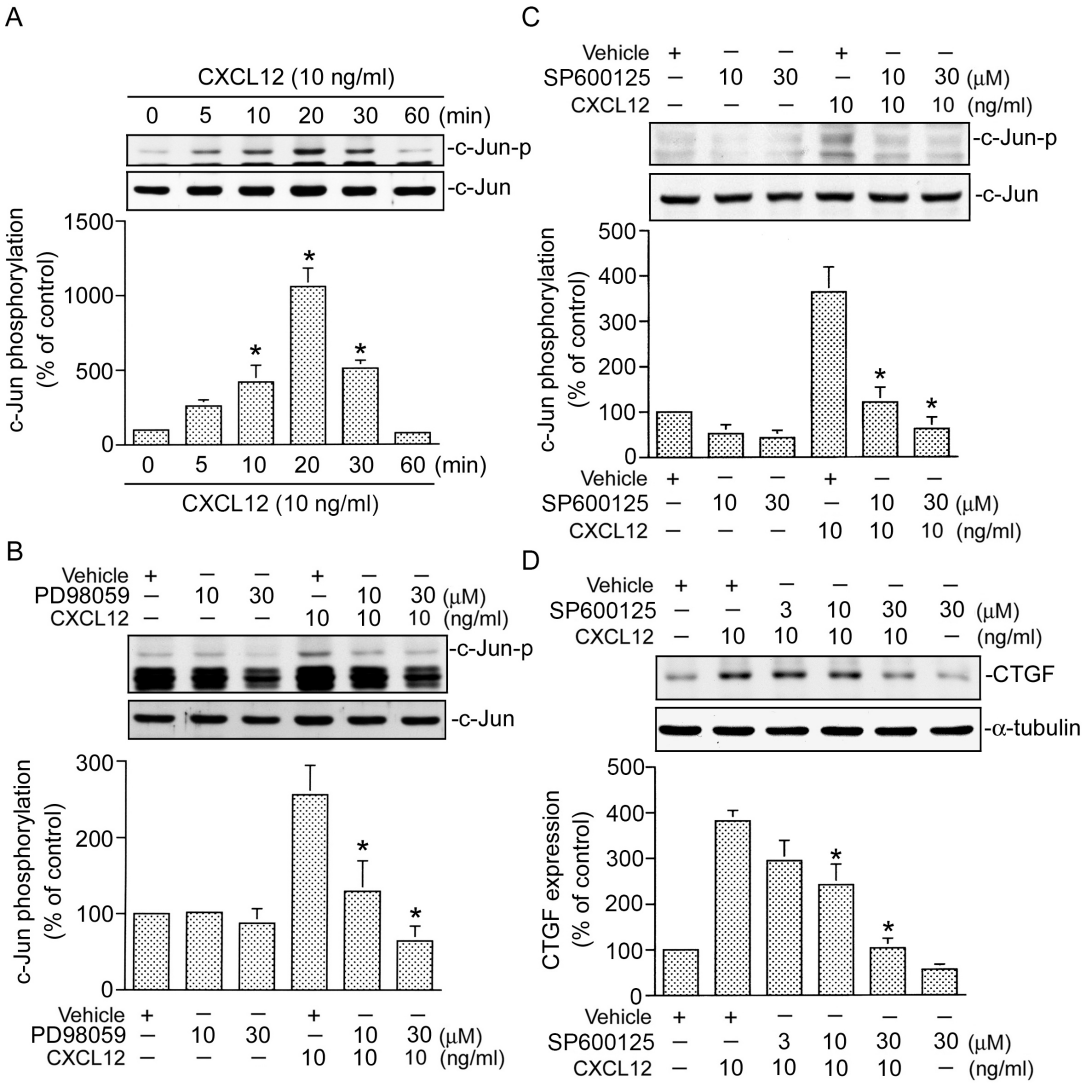
Involvement of extracellular signal-regulated kinase (ERK) and c-Jun N-terminal kinse (JNK) in CXCL12-induced c-Jun phosphorylation or connective tissue growth factor (CTGF) expression in WI-38 cells. A, Cells were treated with CXCL12 (10 ng/ml) for 0∼60 min. Whole-cell lysates were prepared and immunodetected with specific antibodies for phospho-c-Jun Ser 63 or c-Jun. Data are presented as the mean ± S.E. of three experiments. *p<0.05, compared to the control without CXCL12 stimulation. Cells were pretreated with 10 and 30 µM PD98059 (B) or 10 and 30 µM SP600125 (C) for 30 min and then stimulated with CXCL12 (10 ng/ml) for another 20 min. Whole-cell lysates were prepared and immunodetected with specific antibodies for phospho-c-Jun Ser 63 or c-Jun. Data are presented as the mean ± S.E. of three experiments. *p<0.05, compared to CXCL12 treatment. D, Cells were pretreated with SP600125 (3∼30 µM) for 30 min and then stimulated with CXCL12 (10 ng/ml) for another 2 h. Whole-cell lysates were prepared and immunodetected with specific antibodies for the CTGF or α-tubulin. Data are presented as the mean ± S.E. of three experiments. *p<0.05, compared to CXCL12 treatment.

Several studies demonstrated that activation of AP-1 is necessary for CTGF expression [8]. In an attempt to determine whether the AP-1 signaling event is involved in CXCL12-induced CTGF expression, curcumin (an AP-1 inhibitor), was used. As shown in Figure 7A, pretreating cells with curcumin (3 and 10 µM) inhibited CXCL12-induced CTGF expression. In cells treated with 10 µM curcumin, CXCL12-induced CTGF expression was attenuated by 74±9% (n = 3) (Fig. 7A). AP-1 generally consists of heterodimers of c-Jun and c-Fos protein. To confirm whether AP-1 is involved in CTGF expression by CXCL12 stimulation, c-Jun siRNA was used. Figure 7B shows that transfection of cells with c-Jun siRNA (100 nM) also attenuated CXCL12-induced CTGF expression (Fig. 7B). To further confirm results of the protein c-Jun siRNA experiment, we also used c-Jun siRNA to suppress c-Jun protein expression. We found that c-Jun siRNA markedly inhibited c-Jun protein expression (Fig. 7B). These results suggest that AP-1 is involved in CXCL12-induced CTGF expression. Because serine phosphorylation of residue 63 of c-Jun contributes to AP-1 transcriptional activity [21], an antibody specific against phosphorylated c-Jun at Ser63 was used to examine c-Jun phosphorylation. In Fig. 8A, when cells were treated with 10 ng/ml of CXCL12 for various time intervals, c-Jun Ser63 phosphorylation had increased at 5 min, peaked at 20 min, and declined after 30 min of treatment (Fig. 8A). A previous study showed that CXCL12-induced ERK activation mediates c-Jun Ser63 phosphorylation in laryngeal squamous cells [21]. We further examined whether CXCL-12-induced c-Jun phosphorylation occurs through the ERK signal pathway. As shown in Fig. 8B, pretreatment of cells with PD98059 (10 and 30 µM) inhibited CXCL12-induced c-Jun Ser63 phosphorylation in a concentration-dependent manner. (Fig. 8B). This result suggests that activation of ERK is involved in CXCL12-induced c-Jun phosphorylation in WI-38 cells.

### JNK is involved in CXCL12-induced c-Jun phosphorylation and CTGF expression

Our previous studies showed that JNK is involved in thrombin-induced c-Jun phosphorylation and CTGF expression in human lung fibroblasts [8]. To investigate whether JNK also mediates CXCL12-induced c-Jun phosphorylation and CTGF expression, SP600125 (a JNK inhibitor) was used. As shown in Fig. 8C, CXCL12-induced c-Jun phosphorylation at Ser63 was attenuated by SP600125 (10∼30 µM). In cells treated with 30 µM SP600125, CXCL12-induced c-Jun phosphorylation was attenuated by 93±10% (n = 3) (Fig. 8C). We further examined that whether JNK mediates CXCL12-induced CTGF expression and found that SP600125 (3∼30 µM) inhibited CXCL12-induced CTGF expression in a concentration-dependent manner, with 30 µM SP600125 reducing CXCL12-induced CTGF expression by 83±7% (n = 3) (Fig. 8D). These results suggest that in addition to ERK, JNK is also involved in CXCL12-induced c-Jun phosphorylation and CTGF expression.

### CXCL12 induced AP-1 binding to the CTGF promoter in WI-38 cells

**Figure 9:**
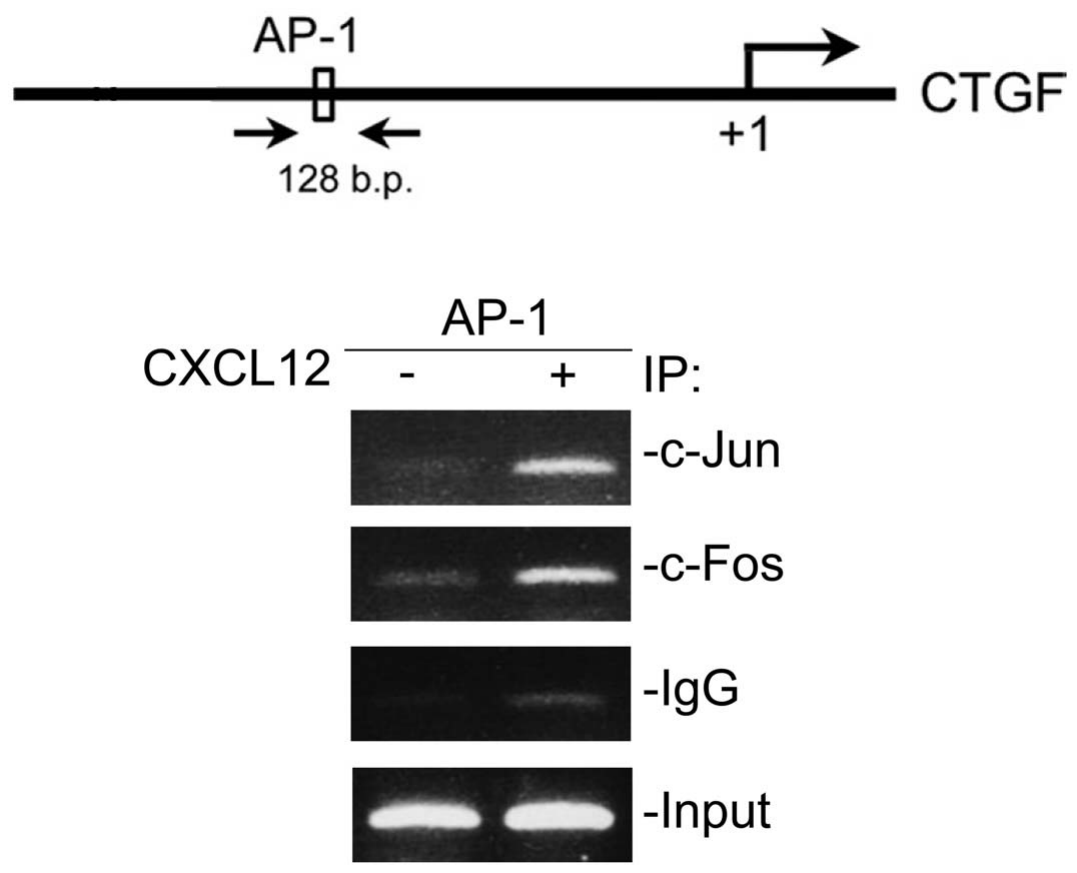
CXCL12 induces activator protein-1 (AP-1) binding to the connective tissue growth factor (CTGF) promoter in WI-38 cells. Schematic diagram of AP-1-binding elements and ChIP primer locations on the CTGF promoter. ChIP primer pairs with a 128-bp PCR product was designed to amplify DNA corresponding to the putative AP-1-binding site. Cells were incubated with 10 ng/ml CXCL12 for 20 min and then cross-linked with formaldehyde at 37°C for another 10 min. Cell lysates were sonicated and prepared for the ChIP assay using antibodies specific for c-Jun and c-Fos as described in “Materials and Methods.” PCR amplification using primers designed against the AP-1-binding site was performed. Equal amounts of soluble cross-linked chromatins present in each PCR were confirmed by the product for input. A rabbit polyclonal IgG antibody was used as a negative control. Typical traces are representative of three experiments with similar results.

The CTGF promoter contains several putative binding sites, including AP-1, which are associated with CTGF expression [8]. To analyze whether the AP-1 subunits, c-Jun and c-Fos, are recruited to the CTGF promoter region in response to CXCL12 stimulation, ChIP experiments were performed on WI-38 cells treated with CXCL12. As shown in Figure 9, treatment with 10 ng/ml CXCL12 for 20 min induced an increase in the recruitment of c-Jun and c-Fos to the AP-1 response element on the promoter region of CTGF (Fig. 9, “IgG”, control IgG; “input”, positive control).

### Involvement of CTGF in CXCL12-induced α-SMA expression

**Figure 10:**
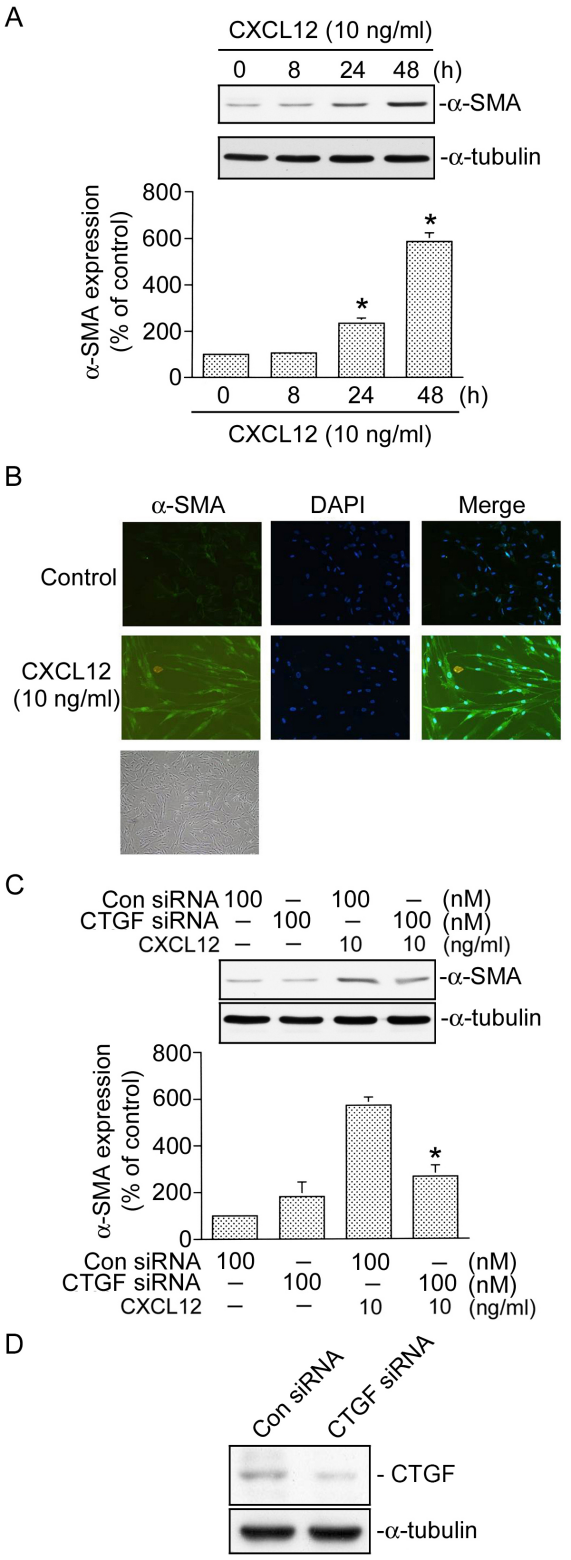
CXCL12 induces α-smooth muscle actin (α-SMA) expression and cell differentiation in WI-38 cells. A, Cells were treated with CXCL12 (10 ng/ml) for 0∼48 h. Whole-cell lysates were prepared and immunodetected with specific antibodies for α-SMA or α-tubulin. Data are presented as the mean ± S.E. of three experiments. *p<0.05, compared to the control without CXCL12 treatment. B, Cells were treated with CXCL12 (10 ng/ml) for 48 h. In an immunofluorescence (200x) analysis, WI-38 cells were stained with α-SMA (green), and nuclei were indicated by DAPI staining (blue). Traces are representative of three experiments with similar results. C, Cells were transfected with control siRNA (Con siRNA 100 nM) or CTGF siRNA (100 nM) for 24 h and then stimulated with CXCL12 (10 ng/ml) for another 48 h. Whole-cell lysates were prepared and immunodetected with specific antibodies for α-SMA or α-tubulin. Data are presented as the mean ± S.E. of three experiments. *p<0.05, compared to CXCL12 treatment. D, Cells were transfected with control siRNA (Con siRNA 100 nM) or CTGF siRNA (100 nM) for 24 h. Whole-cell lysates were prepared and immunodetected with specific antibodies for the CTGF or α-tubulin. Traces represent results from three independent experiments.

A previous report demonstrated that CXCL12 induced breast carcinoma-associated fibroblast differentiation into myofibroblasts [40]. Next, we examined whether CXCL12 induced lung fibroblast differentiation. Fibroblast differentiation induced by CXCL12 is characterized by the expression of α-SMA. As shown in Fig. 10A, incubation of cells with CXCL12 (10 ng/ml) for different time intervals induced α-SMA expression in a time-dependent manner, with a maximum effect at 48 h of CXCL12 treatment (Fig. 10A). Similarly, in an immunofluorescence staining experiment, we found that CXCL12 (10 ng/ml) induced increases in α-SMA expression and myofibroblastic phenotype formation (green fluorescence) (Fig. 10B). A previous study indicated that CTGF contributes to the expression of α-SMA and is involved in the development of pulmonary fibrosis including fibrocyte and fibroblast differentiation [5]. To further determine whether CXCL12-induced CTGF expression contributes to lung fibroblast differentiation, CTGF siRNA was used. The siRNA experiments revealed that CTGF siRNA (100 nM) inhibited CXCL12-induced α-SMA expression by 82±10% (n = 3) (Fig. 10C). To further confirm results of the CTGF siRNA experiments, we also used CTGF siRNA to suppress CTGF protein expression. As shown in Fig. 10D, CTGF siRNA markedly inhibited CTGF expression in WI-38 cells (Fig. 10D). Taken together, these results indicated that induction of CTGF expression contributes to CXCL12-induced α-SMA expression and fibroblast differentiation in WI-38 cells.

### CXCR4 mediates CXCL12-induced actin stress fiber formation in WI-38 cells

**Figure 11:**
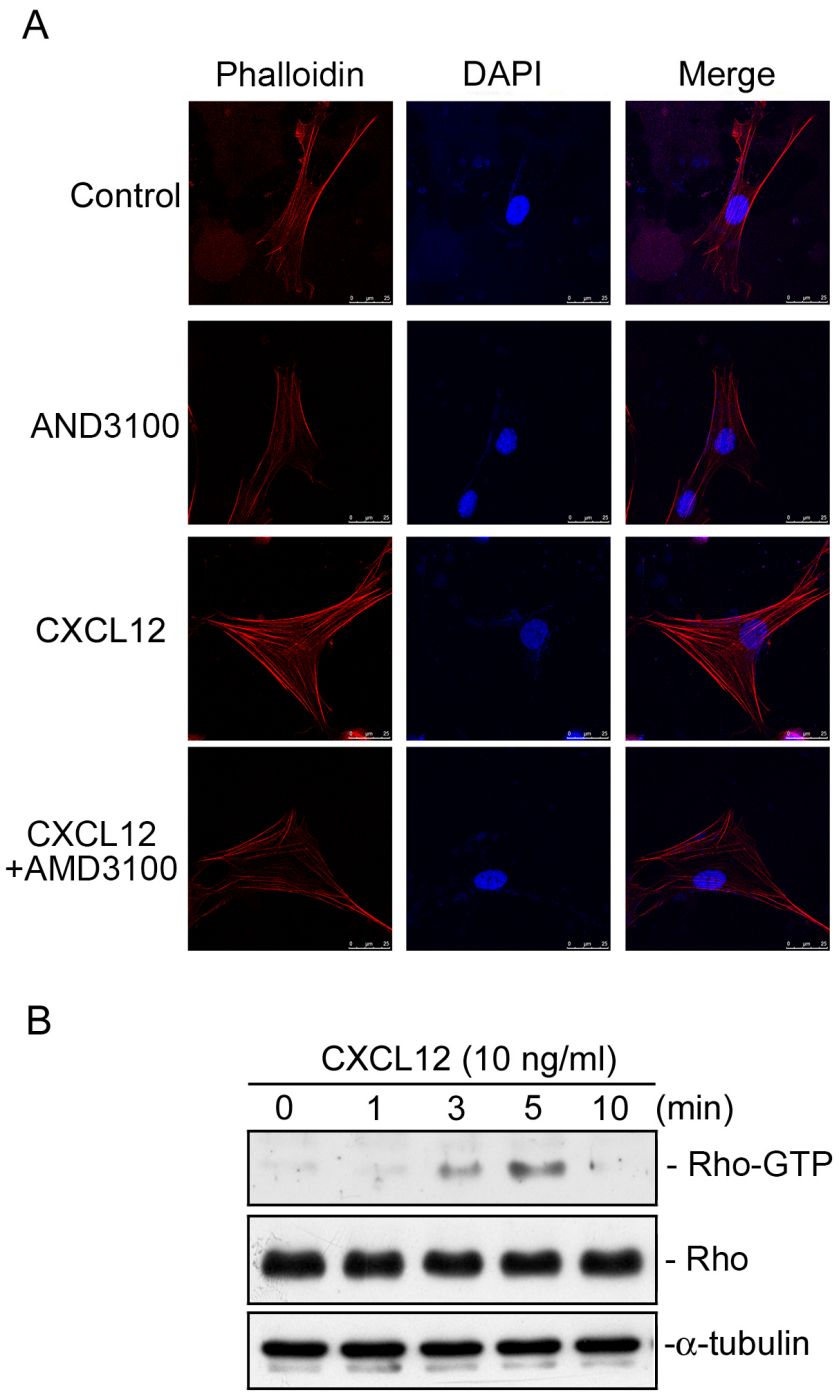
The CXCL12 induces actin stress fiber formation and Rho activity in WI-38 cells. A, Cells were pretreated with ADM3100 (10 µM) for 20 min, and then stimulated with CXCL12 (10 ng/ml) for another 24 h. In a confocal microscopic analysis, cells were stained with rhodamine-phallodin (red) and nuclei were indicated by DAPI staining (blue). Scale bar, 25 µm. Traces are representative of three experiments with similar results. B, Cells were treated with CXCL12 (10 ng/ml) for different time intervals. Whole-cell lysates were then immunoprecipitated with Rhotekin RBD-agarose. The Rho activity assay is described in “Materials and Methods”. Rho and α-tubulin protein levels were determined in total lysates by a Western blot analysis as the loading control. Immunoblots are representative of three experiments with similar results.

Several reports indicated that the CXCL12/CXCR4 signaling axis regulates cell migration and actin stress fiber formation [20], [41]. Actin stress fibers are responsible for cell traction and ECM reorganization [42]. Next, we investigated whether the CXCL12/CXCR4 axis regulates actin stress fiber formation in human lung fibroblasts. In a confocal microscopic analysis, we found that CXCL12 (10 ng/ml) induced an increase in actin stress formation, which was inhibited by 10 µM AMD3100 (Fig. 11A). Many studies revealed stress fiber formation is regulated by activation of Rho by various stimuli [42], [43]. We further elucidated whether CXCL12 can induce Rho activation in WI-38 cells. Figure 11B shows that treatment of WI-38 cells with CXCL12 (10 ng/ml) induced an increase in Rho activity in a time-dependent manner. Rho activity had increased at 3 min and peaked at 5 min after CXCL12 treatment (Fig. 11B). These results suggest that the CXCL12/CXCR4 axis signaling pathway may play a critical role in actin stress fiber formation in WI-38 cells.

## Discussion

There is evidence that increased CTGF protein levels are found in most tissues with fibrotic diseases, and CTGF plays a critical role in pulmonary fibrosis. Previous studies indicated that CTGF modulates pulmonary fibrosis via fibroblast proliferation, fibrocyte differentiation into fibroblasts/myofibroblasts, and ECM synthesis [5], [44]. Our previous study indicated that ET-1-induced CTGF expression contributes to pulmonary fibrosis [5]. A large body of evidence has accumulated which suggests that CXCL12 and its receptor, CXCR4, participate in pulmonary fibrosis [17], [18]. Previous reports indicated a critical role of the CXCL12/CXCR4 axis in fibrocyte migration in the lungs and its contribution to pulmonary fibrosis [45], [46]. Moreover, Phillips et al. demonstrated that treatment with a neutralizing antibody of CXCL12 improved bleomycin-induced pulmonary fibrosis [46]. Recently, Makino et al. showed that administration of the CXCR4 antagonist, AMD3100, significantly reduced bleomycin-induced pulmonary fibrosis in mice [18]. Therefore, the CXCL12/CXCR4 axis and CTGF play important roles in pulmonary fibrosis. The findings of our study demonstrated that CXCL12 activates CXCR4, which in turn initiates Rac1/ERK, JNK, and AP-1 activation, and ultimately induces CTGF expression in human lung fibroblasts. Moreover, overexpression of CTGF mediates CXCL12-induced α-SMA expression. According to the present results, it may be significant to understand which signal pathway is involved in CXCL12-induced CTGF expression in lung fibroblasts.

Activation of CXCR4 by CXCL12 plays a key role in regulating diverse cellular responses, including cell migration, chemotaxis, and expressions of inflammatory mediators through stimulating downstream signaling such as Rac1 [29]–[31]. Rac1 was demonstrated to play an important role in promoting Jurkat T cell migration [20]. Rac was shown to be required to activate transcription factors, including AP-1, and mediates gene expression by various stimuli [47]. Chen et al. [48] demonstrated that activation of Rac1 was required for CTGF expression caused by stretch stimulation in mesangial cells. In addition, activation of Rac1 mediated CTGF expression in dermal fibroblasts cultured from lesional areas of scleroderma patients [49]. Because Rac1 was reported to be a downstream effector of the CXCL12/CXCR4 axis, we raised the question of whether Rac1 plays a role in CXCL12-induced CTGF expression. In this study, we found that transfection of WI-38 cells with a Rac1 dominant negative mutant (RacN17) inhibited CXCL12-induced CTGF expression. Moreover, CXCL12 induced an increase in Rac1 activity in lung fibroblasts. These results indicate that Rac1 is required for CTGF expression caused by CXCL12 stimulation in lung fibroblasts.

Mounting evidence shows that MAPK cascades are activated through Ser/Thr kinase groups that participate in intracellular signal transduction and mediate tissue fibrosis [33]. Additionally, ERK was previously implicated in a signal pathway relevant to CTGF expression [50]. A previous report showed that the ERK signaling pathway is involved in TGF-β-induced CTGF expression in hepatic stellate cells [50]. Another report showed that a MEK inhibitor (PD98059) abrogated *Pasteurella multocida* toxin-induced CTGF protein expression in Swiss 3T3 cells [51]. Moreover, a previous report showed that CXCL12-induced sonic hedgehog expression depends on ERK activation in pancreatic cancer cells [52]. In this study, we present results of the role of ERK in CXCL12-induced CTGF expression in lung fibroblasts. We found that a MEK inhibitor (PD98059) inhibited CXCL12-induced CTGF expression in WI-38 cells. Moreover, treatment of cells with CXCL12 induced ERK phosphorylation. These results suggest that ERK activation is involved in CXCL12-induced CTGF expression in human lung fibroblasts. Our previous study indicated that JNK participates in thrombin-induced CTGF expression in human lung fibroblasts [8]. In this study, we provide data that confirm the role of JNK in CXCL12-induced CTGF expression. We found that SP600125 (a JNK inhibitor) attenuates CXCL12-induced CTGF expression. This result suggests that JNK is also involved in CTGF expression with CXCL12 stimulation. A previous report showed that prolactin-induced ERK activation is dependent on Rac1 in breast cancer cells [53]. Wang et al. reported that inhibition of Rac1 activity leads to dysfunction of ERK in keratinocytes [54]. Williams et al. demonstrated that TNF-α-induced ERK activation and cytokine production are mediated by the Rac1 pathway in HeLa cells [55]. In this study, we found that CXCL12-induced ERK phosphorylation at the Tyr204 residue was markedly attenuated by RacN17. These results emphasize that the Rac1 signaling pathway plays a role in thrombin-induced ERK activation in human lung fibroblasts.

The transcription factor, AP-1, usually consists of heterodimers of c-Jun and c-Fos proteins that mediate several gene expressions in different cell types [56]. The promoter region of the human *CTGF* gene contains several transcription factor-binding sites including STAT, SMAD, BCE-1, NF-κB, Sp1, Ets-1, and AP-1 [26]–[28]. Of these, the AP-1 site was reported to be critical for induction of CTGF transcription [8], [57]. Our previous study showed that AP-1 is involved in thrombin-induced CTGF expression in human lung fibroblasts [8]. Recchia et al. pointed out that activation of AP-1 is associated with ET-1-induced CTGF expression in cardiomyocytes [58]. In this study, we confirmed the role of AP-1 in CXCL12-induced CTGF expression in human lung fibroblasts. We found that treatment of WI-38 cells with curcumin, an AP-1 inhibitor, and c-Jun siRNA both attenuated CXCL12-induced CTGF expression. We also found that CXCL-12 induced an increase in c-Jun phosphorylation. Moreover, results from the ChIP experiment indicated that CXCL12 stimulation caused c-Jun and c-Fos to directly bind to the CTGF promoter region. Our results are consistent with a previous study which found that the AP-1 transcription factor participates in serum-induced CTGF expression in keloid fibroblasts [57]. Several studies indicated that 1,2-naphthoquinone- and ET-1-induced AP-1 activation depends on ERK activation in human lung epithelial cells and cardiomyocytes [58], [59]. A previous study indicated that ERK activation is involved in c-Jun phosphorylation in aryngeal and hypopharyngeal squamous cell carcinomas [21]. Our previous study showed that JNK is involved in thrombin-induced c-Jun phosphorylation in human lung fibroblasts [8]. In this study, we found that PD98059 and SP600125 both inhibited the CXCL12-induced increase in c-Jun phosphorylation. This result suggests that ERK and JNK are involved in CXCL12-induced c-Jun phosphorylation in WI-38 cells.

Several reports indicated that induction of CXCL12 plays an important role in pulmonary fibrosis [11], [12]. Moreover, overexpression of the CTGF participates in the pathogenesis of pulmonary fibrosis [26]. Treatment with an anti-CTGF antibody was demonstrated to markedly inhibit the severity of pulmonary fibrosis in a mouse model [60]. The CTGF is considered a key protein in TGF-β-, angiotensin II-, and ET-1-induced tissue fibrosis [5], [61], [62]. A previous study indicated that angiotensin II can induce fibrocyte differentiation, which induces high CTGF levels and contributes to myocardial fibrosis [62]. A previous report showed that CXCL12 can induce bone marrow-derived cell differentiation into cardiomyocyte phenotypes in vitro [16]. A recent study showed that CXCL12 is involved in breast carcinoma-associated fibroblast differentiation into myofibroblasts during tumor progression [40]. However, whether CXCL12 can induce human lung fibroblast differentiation into myofibroblasts is still unclear. In this study, we found that CXCL12 induced the expression of α-SMA, a differentiation marker, in human lung fibroblasts and produced myofibroblastic phenotype formation. Moreover, we also demonstrated a role of the CTGF in mediating CXCL12-induced α-SMA expression in WI-38 cells. We found that CTGF siRNA significantly inhibited CXCL12-induced α-SMA expression. This is consistent with findings of our previous report that the CTGF mediates ET-1-induced fibrocyte differentiation into fibroblasts/myofibroblasts [5]. Taken together, these results suggest that CTGF contributes to CXCL12-induced fibroblast differentiation.

A growing body of evidence has demonstrated that the CXCL12/CXCR4 axis and Rho signaling pathway play critical roles in cell migration, chemotaxis, tumor metastasis, and actin stress fiber formation [20], [43], [63]. A previous study showed that CXCL12/CXCR4 axis signaling induces actin stress fiber formation, and regulates tumor spread and metastasis [63]. Tan et al. [2006] indicated that activation of Rho is involved in CXCL12/CXCR4-mediated cell migration [20]. Another study demonstrated that growth factor-induced actin stress fiber formation depends on Rho activation in Swiss 3T3 fibroblasts [64]. In this study, we further demonstrated the role of CXCR4 in CXCL12-induced actin stress fiber formation. We found that a CXC4 inhibitor (AMD3100) inhibited CXCL12-induced actin stress fiber formation in human lung fibroblasts. Moreover, treatment of cells with CXCL12 caused Rho activation. These results suggest that the CXCL12/CXCR4 axis signaling pathway plays a critical role in actin stress fiber formation in WI-38 cells.

**Figure 12:**
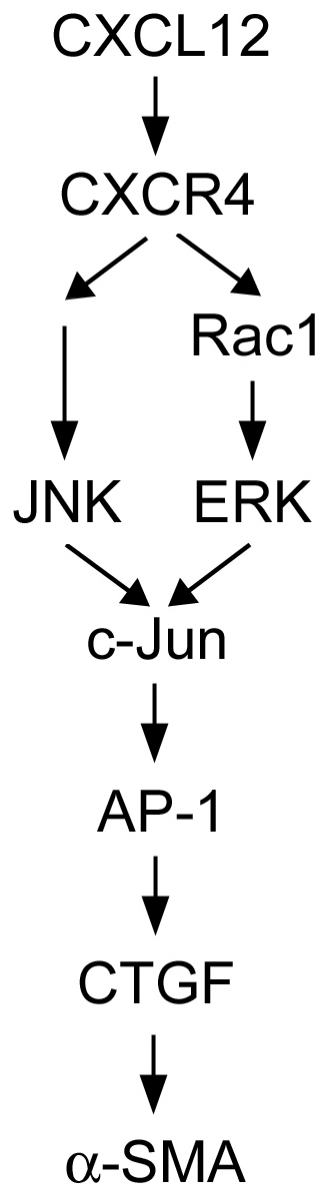
Schematic summary of the signal transduction pathway by which CXCL12 induces connective tissue growth factor (CTGF) expression in human lung fibroblasts (WI-38). CXCL12 acts on CXCR4 to activate the Rac/extracellular signal-regulated kinase (ERK) and c-Jun N-terminal kinase (JNK) signaling pathways, which in turn initiates activator protein-1 (AP-1) activation, and ultimately causes CTGF expression. Moreover, CTGF mediates CXCL12-induced α-smooth muscle actin (α-SMA) expression in human lung fibroblasts.

In conclusion, the current study showed that CXCL12, acting through CXCR4, activates the Rac/ERK and JNK signaling pathways, which in turn initiates c-Jun phosphorylation, recruits c-Jun and c-Fos to the CTGF promoter, and ultimately induces CTGF expression in human lung fibroblasts. The CXCL12/CXCR4 axis signaling pathway also contributes to actin stress fiber formation. Moreover, induction of CTGF expression mediates CXCL12-induced α-SMA expression. Figure 12 is a schematic representation of the signaling pathway showing that CXCL12 induces CTGF expression via the CXCR4, Rac1/ERK, JNK, and AP-1 signaling pathways in human lung fibroblasts. Our results present a signaling pathway associated with CXCL12 and the profibrotic protein, CTGF, and provide support for the development of therapeutic strategies to reduce pulmonary fibrosis caused by CXCL12.
